# The role of fructose-1,6-bisphosphatase 1 on regulating the cancer progression and drug resistance

**DOI:** 10.1007/s12672-025-02112-2

**Published:** 2025-03-18

**Authors:** Mengmeng Wang, Xiaoju Huang, Dan Zhang, Yisan Liu, Pian Liu

**Affiliations:** 1 Cancer Center, Union Hospital, Tongji Medical College, Huazhong University of Science and Technology, Wuhan, 430022 China; 2 Institute of Radiation Oncology, Union Hospital, Tongji Medical College, Huazhong University of Science and Technology, Wuhan, 430022 China; 3 Hubei Key Laboratory of Precision Radiation Oncology, Wuhan, China; 4 Department of Urology, People’s Hospital of Cili, Cili, 427200 Hunan China

**Keywords:** FBP1, Cancer, Drug resistance, Small molecule drugs

## Abstract

Fructose-1,6-bisphosphatase 1 (FBP1) is the enzyme that limits the process of gluconeogenesis as it facilitates the hydrolysis of fructose-1,6-bisphosphate(F-1,6-BP) to produce fructose-6-phosphate(F6P) and inorganic phosphate. Gluconeogenesis is the production of glucose from small carbohydrate substrates. The gluconeogenic process is typically suppressed in cancer because it inhibits glycolysis. Apart from its involvement in cellular glucose metabolism, FBP1 also plays a role in gene transcription, mRNA translation and stability regulation, and the immune microenvironment of tumors. Because of its multifaceted functions, the mechanisms by which FBP1 is involved in tumor development are complex. Moreover, FBP1 deficiency is associated with radiation and chemotherapy resistance and poor prognosis in cancer patients. Restoration of FBP1 expression in cancer cells is expected to hold promise for cancer therapy. However, up to now few reviews have systematically summarized the important functional mechanisms of FBP1 in tumorigenesis and the small molecule compounds that restore FBP1 expression. Therefore, this article addresses the question “How does FBP1 contribute to cancer progression, and can targeting FBP1 be a potential therapeutic approach?” by summarizing the effects of FBP1 on cancer development and progression as well as its mediated drug resistance and the future clinical applications of potential small molecule modulators targeting FBP1.

## Introduction

FBP1 is the first FBPase to be discovered, and it is expressed predominantly in gluconeogenic tissues like the kidney and liver. It is situated on chromosome 9q22.32 and comprises seven exons encoding 338 amino acids [1]. FBP1 is a homotetrameric protein with each subunit having a molecular weight of approximately 37 kDa, with three structural domains, the central structural domain (amino acid residues 107–447), the carboxyl terminus of FBP (FBPC, amino acids 488–644), and the amino terminus of FBP (FBPN: amino acid). FBP1 has two conformations: the active state (R, loose) and the inactive state (T, tight) [2], and conformational changes can occur after binding with different ligands. Ligands such as metal ions (Mg^2+^) and FDP stabilize the activated conformation of FBPase. Whereas, when the physiological inhibitor AMP binds to FBPase, the enzyme subunits rotate 15° clockwise, affecting the Mg^2+^ binding site and interfering with the active site of the enzyme, thus inactivating it [3]. FBP1 is located in the cytoplasm, nucleus, and cellular exosomes, and plays an important role in regulating the biological functions of cells.

Disturbances in cellular metabolic activity are at the root of tumorigenesis, with alterations in energy metabolism being a fundamental feature of cancer [4]. The amount of glucose uptake by tumor cells increases to accommodate the unrestricted growth and division of tumor cells. Large amounts of ATP are produced in a short-circuited fashion because the enzyme lactate dehydrogenase (LDH) is preferentially converted to lactate even when there is enough oxygen present for the mitochondria to metabolize glucose. Tumor cells predominantly obtain their energy by aerobic glycolysis (i.e., the Warburg effect) [5, 6] characterized by a sharp rise in the rate of glycolysis and lactate generation. FBP1, an enzyme that limits the process of gluconeogenesis, hampers the Warburg effect, thus inhibiting tumor development.

In addition to regulating glucose metabolism, FBP1 also regulates cell proliferation, migration, differentiation and apoptosis by regulating DNA transcription and mRNA translation and stability [7, 8]. FBP1 was initially identified as a DNA-binding protein that transcriptionally regulates the expression of the oncogene c-myc [7], and it was later found that FBP1 can regulate the expression of a wide range of oncogenes and oncostatic genes at both the transcriptional and post-transcriptional levels [9]. Moreover, FBP1 can affect tumor immune responses by regulating the number and activity of NK cells and the function of T cells [10]. FBP1 also inhibits multiple tumor immune escapes by down-regulating the expression of PD-L1 [11].

Due to its complex biological functions, FBP1 has emerged as a key target in tumor studies in the past few decades. In addition to FBP1 deletion significantly affecting the progression of hepatocellular carcinoma [12] and renal carcinoma [13], it is also down-regulated in gastric [14], pancreatic [15], colorectal [16], breast [17], prostate [18], bile duct [19], esophageal [20], lung [21], and ovarian cancers [22]. Therefore, to address the question, “How does FBP1 promote cancer progression, and could targeting FBP1 be a potential therapeutic approach?”, we review the specific mechanisms of FBP1 in regulating tumor progression and drug resistance and explored the application of FBP1 as a new diagnostic biomarker and therapeutic target in tumor therapy.

## Biological functions of FBP1 Fig. 1

**Fig. 1:**
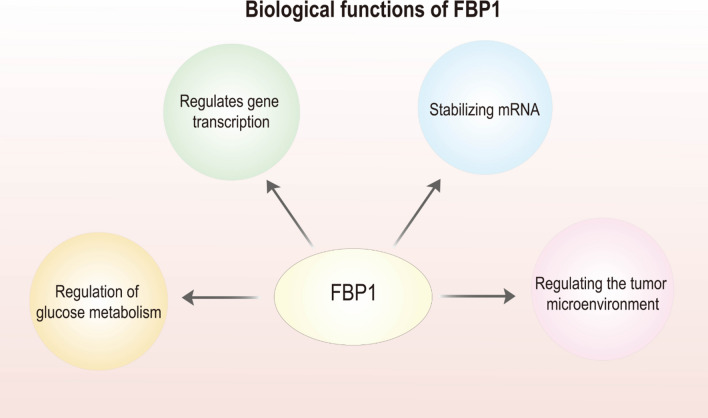
Biological functions of FBP1

### Regulation of glucose metabolism

FBP1 in the cytoplasm is an important gluconeogenic enzyme that hydrolyzes F-1,6-BP to F6P during gluconeogenesis [23]. Other key enzymes in the gluconeogenesis pathway include pyruvic carboxylase (PC), phosphoenolpyruvate carboxykinase (PEPCK), and glucose-6-phosphatase (G6Pase). FBPase acts in the second step of gluconeogenesis and is the rate-limiting enzyme [24]. Based on its position in the gluconeogenesis pathway, it is able to inhibit gluconeogenesis of all relevant substrates; therefore, FBPase is essential for the synthesis, export, and control of endogenous glucose in the body.Gluconeogenesis primarily occurs in the liver, whereas the gluconeogenesis capacity of the kidney is weak but can be enhanced during prolonged starvation. Gluconeogenesis in the liver primarily regulates blood glucose during starvation or exercise and also restores the synthesis of hepatic glycogen reserves during the initial period of feeding after starvation. On the other hand, gluconeogenesis in the kidney maintains acid–base homeostasis during prolonged starvation. In addition, FBPase synergizes with 6-phosphofructokinase (PFK), the rate-limiting enzyme in glycolysis, and determines the dynamic conversion between F6P and FDP in the cell, as well as the dynamic balance of glucose between enzymolysis and xenobiosis [25].

### Regulation of gene transcription

FBP1 can function as a transcriptional regulator in cells, and its nuclear localization is mainly driven by three nuclear localization signals (NLS) in its N-terminal, middle.

and C-terminal structural domains  [26]. FBP1 is a bifunctional protein that activates some genes and represses others.FBP1 uses a set of four KH motifs to destabilize the FUSE double helix and binds sequence-specifically to the non-coding chain to exert transcriptional activation [27]. The transcriptional activation triggered by TFIIH is selectively suppressed by FIR. FIR binds to the central DNA-binding domain of FBP1 and interacts with the TFIIH complex to exert transcriptional repression [28]. The FBPC activate transcription when fused to the GAL4 DNA-binding domain. On the other hand, expression triggered by FBPC and some heterologous activators is suppressed by FBPN [29].The repressive and activating structural domains of FBP are possibly effectively coordinated; for instance, FBP may upregulate transcription when it is required for c-Myc expression, but FBP1 exerts transcriptional repression if c-myc expression is too high.

### Stabilization of mRNA

FBP1 also functions as an RNA-binding protein, and its KH domain is essential for protein-RNA binding [30]. The four KH domains found in each member of the FBP family have the ability to bind not only to RNA but also to single-stranded DNA. FBP1 binds to the 3′-UTR site of mRNAs from multiple genes; this site is known to cause mRNA instability.It has been shown that FBP1 interacts specifically with the 3' UTR of nuclear phosphoprotein (NPM/B23) to inhibit its translation [31]. FBP1 binds to the 5′-untranslated region of p27 RNA through four K homologous motifs in the central structural domain, and the 5′-untranslated region (5′-UTR) of p27 mRNA contains an internal ribosomal entry site (IRES), which may facilitate p27 synthesis under certain circumstances. FBP1 has been shown to bind directly to the 5′-UTR of human p27 and promote IRES activity [32].

### Regulating the tumor microenvironment

A mixed tumor microenvironment (TME) of epithelial and mesenchymal stem cells (MSCs), vascular and lymphatic vessels, and inflammatory and infiltrating immune cells gives rise to tumors. There is growing evidence that an immunosuppressive TME is associated with tumorigenesis, progression, and immune escape [33]. NK cells are included among effector cells; these lymphocytes show cytotoxic activity against cancer cells and microbial infections through the release of cytokines and chemokines and thus exhibit great immunotherapeutic potential. The study showed that gastric cancer mesenchymal stem cells (GCMSCs) can inhibit NK cell function by upregulating the expression of FBP1 in NK cells and decreasing glucose metabolism in NK cells [10]; similarly in lung cancer, FBP1 impairs NK cell viability by inhibiting glycolysis in NK cells [34]. Moreover, targeted inhibition of FBP1 restored NK cell function in gastric cancer and lung cancer models, which opens up new avenues for NK-cell-based immunotherapy. Liu et al. found that an FBP1-deficient liver exhibits reduced numbers of NK cells and accelerated tumorigenesis and hepatic FBP1 depletion promotes hepatocellular carcinoma-related immune remodeling [35]. Thus FBP1 may exert different immune effects in different cancer types by regulating the function of NK cells. In addition, FBP1 can inhibit tumor immune escape by suppressing PD-L1 expression [11].FBP1 is also involved in mediating inflammatory responses. 1α, 25(OH)2D3 inhibits cytokine generation in γδ T cells via the FBP1/Akt/p38 MAPK pathway. Thus, γδ T cells producing cytokine IL-17 (γδT17) have a vital function in the regulation of infection, inflammation, cancer, and insulin resistance [36, 37]. The latest research on the effect of gefitinib in the progression of lung adenocarcinoma (LUAD) discovered that a risk model constructed around its target genes FBP1, SBK1, and AURKA was associated with a poor prognosis and was strongly connected to the immune microenvironment [38], although the exact mechanism remains unclear.

## Mechanisms of FBP1-mediated tumor suppression

In most cases, FBP1 is poorly expressed in cancers as an anti-oncogene, and its low expression predicts a bad prognosis for certain types of cancer [39]. In addition to inhibiting tumor growth by antagonizing glycolysis, FBP1 inhibits cell proliferation through a series of non-glycolytic pathways; for instance, by acting as a transcriptional regulator of DNA transcription, or directly binding to mRNA to regulate mRNA translation and stability. He et al. demonstrated that FBP1 regulates fatty acid metabolism [20], and in recent years, FBP1 was found to also act as a protein phosphatase to regulate cancer progression. Therefore, restoration of FBP1 expression results in anticancer effects.

### FBP1 antagonizes glycolysis to inhibit tumor progression

FBP1 is an important regulatory enzyme in gluconeogenesis. FBP1 is deficiently expressed in HCC, gastric cancer, colon cancer, and nasopharyngeal carcinoma, accelerating glucose uptake and glycolysis [40]. Research has reported that elevated levels of FBP1 are linked to a better prognosis in various tumor diseases including renal cancer, gastric cancer, and lung cancer, suggesting that FBP1 acts an essential part in cancer glucose metabolism. Table 1

**Table 1:** FBP1 antagonizes glycolysis to inhibit tumor progression

Cancer types	Molecular mechanisms	References
Pancreatic cancer	NPM1/FBP1	[15]
	CBX3/FBP1	[41]
	CDK4/6-E2F1/MAGED1/FBP1	[42]
Gastric cancer	Snail/FBP1	[43]
	Ras/ NF-κB/ FBP1	[44]
Colorectal cancer	FOXC1/FBP1	[16]
	miR-145/ AF9/FBP1	[50]
Breast cancer	HMGB2/FBP1	[17]
Glioma	GBE1/ NF-κB/FBP1	[45]
Lung adenocarcinoma	HIF1α/GBE1/NF-κB/FBP1	[46]
Renal carcinomas	miR-24-1/ FBP1	[51]
	EZH2/FBP1	[48]
Hepatocellular carcinoma	HDAC1、 HDAC2/FBP1	[47]
	MAGE-TRIM28/FBP1	[49]
	EZH2/ FBP1	[48]
Prostate cancer	PTEN/ FBP1	[18]
Breast cancer, Renal cancer	FBP1/HIF-1α	[55, 56]

#### The overexpression of several oncogenes in cancer cells inhibits FBP1 expression at the transcriptional level, thereby increasing cell glycolysis

NPM1, a functionally complex nucleolar protein, is upregulated in pancreatic ductal adenocarcinoma and inhibits FBP1 expression at the transcriptional level, promoting the Warburg effect [15]. Chromobox protein homolog 3 (CBX3) is a heterochromatin protein that is upregulated in various tumors and can epigenetically regulate crucial genes for cancer development and growth. In pancreatic cancer, CBX3 downregulates FBP1 expression and promotes glycolysis [41], while the CDK4/6-E2F1 pathway increases MAGED1 expression and promotes FBP1 degradation, and the CDK4/6 inhibitor PD0332991 eliminates the Warburg effect [42]. In gastric cancer, Snail inhibits the FBP1 expression at the transcriptional level to enhance glycolysis during the epithelial-mesenchymal transition [43]. Moreover, the NF-kappaB downstream of Ras downregulates FBP1 at the transcriptional level to enhance the Warburg effect and promote gastric cancer progression [44]. The abnormal expression of FOX transcription factors has a crucial role in carcinogenesis. FOXC1 exhibits an enhanced expression in colorectal cancer; it directly binds to the promoter region of the FBP1 gene and inhibits transcriptional activity, promoting colorectal cancer growth by enhancing the Warburg effect [16]. High mobility group box (HMGB) is a protein that is widely expressed in humans and has been found to be overexpressed in a variety of tumors, binding to DNA to regulate gene transcription. Fu et al. [17] found that HMGB2 expression is greater in the nucleus of breast cancer than in the surrounding normal breast cells. HMGB2 promotes LDHB expression and inhibits FBP1 expression, which in turn promotes breast cancer growth via boosting the Warburg effect. GBE1 can be expressed as an oncogene in various tumors. In gliomas, GBE1 reduces the expression of FBP1 through the NF-κB pathway. This shifts glioma cells' glucose metabolism to glycolysis, boosting the Warburg effect and driving glioma growth [45]. In LUAD, hypoxia induces the upregulation of HIF1α, which binds to the promoter of GBE1, in turn upregulating GBE1 and inhibiting FBP1 expression through promoter methylation induced by the NF-κB pathway. FBP1 downregulation not only leads to an enhanced HIF1α expression in LUAD cells and an increased glucose uptake but also triggers the transition to anaerobic glycolysis [46]. In HCC cells, HDAC1 and HDAC2 limit FBP1 transcription by decreasing H3K27 acetylation in the FBP1 enhancer region. Pharmacological inhibition of HDAC and genetic reduction of HDAC1 and HDAC2 increase the level of H3K27Ac and hinder FBP1 inhibition [47]. EZH2 is an essential part of the Polycomb repressive complex 2 (PRC2), which inhibits FBP1 expression by inducing H3K27 trimethylation (H3K27me3). FBP1 interacts with EZH2 to inhibit EZH2’s methyltransferase activity in a feedback manner, leading to a further reduction of FBP1. In turn, reduced FBP1 levels increases glycolysis and leads to the progression of HCCs and renal carcinomas [48].

#### The deletion of anti-oncogenes in tumor cells promotes tumor glycolysis by downregulating FBP1

Song et al. found that PTEN loss enhances the Warburg effect and the development of prostate cancer by causing FBP1 degradation [18]. In HCC, the MAGE-TRIM28 combination accelerates the Warburg effect and the development of HCC by degrading FBP1 [49]. miR-145 targets ASTM A and FBP1 degradation to promote tumor glycolysis. Moreover, miR-145 targets the 3' untranslated region of AF9 to inhibit AF9 expression. Gluconeogenic genes, such as phosphoenolpyruvate carboxykinase 2 (PCK2) and FBP1, are less expressed when AF9 is deleted, promoting glucose depletion and colorectal cancer [50]. Reduced level of enhancer-associated miR-24-1 leads to inactivation of FBP1, and depletion of FBP1 further promotes the Warburg effect, ultimately contributing to the development of RCC [51].

#### FBP1 plays an important role in the energy metabolism of cancer cells by regulating certain glycolysis-related genes

Hypoxia-inducible factor 1α (HIF-1α) is a transcription factor that promotes cellular glycolysis, resulting in the Warburg effect [52]. HIF-1α is upregulated in many human cancers including colon, breast, gastric, lung, skin, ovarian, pancreatic, prostate, and kidney cancers [53]; thus, inhibiting HIF-1α may result in anticancer effects by regulating cell growth, invasion, and migration [54]. In breast cancer cells, FBP1 and HIF-1α are oppositely expressed. Under hypoxic conditions, FBP1 overexpression suppresses the mRNA expression of the HIF-1α target genes PDK1, LDHA, GLUT1, and VEGF, inhibiting tumor growth, migration, and glycolysis in BLBC [55]. Ning et al. found that FBP1 inhibits the action of the HIF situated in the nucleus, which may help understand the mechanism of ccRCC tumorigenesis [56].

### FBP1 inhibits tumor proliferation and migration through non-glycolytic pathways

#### FBP1 acts as a protein phosphatase to regulate cancer progression

Metabolic enzymes do not have just one function or catalyze only one step of the reaction, but under the joint action of oncogenes and the unique TME, they can perform many non-classical functions and synergistically regulate tumor development. FBP1 can also function as a protein phosphatase to play complex biological roles in tumors Table 2.

**Table 2:** FBP1 acts as a protein phosphatase to regulate cancer progression

Cancer types	Molecular mechanisms	Function	References
Hepatocellular carcinoma Renal cell carcinoma	FBP1/histoneH3/PPARα	Inhibit β-oxidation and tumor growth	[57]
	FBP1/TERT	Inhibit telomerase activity	[58]
Colorectal cancer	FBP1/IκBα/ NF-κB	Promote of anti-tumor immunity	[59]

Glucose deprivation occurring in normal hepatocytes induces PERK-dependent FBP1 S170 phosphorylation, converting the FBP1 tetramer into a monomer and exposing the NLS, ultimately leading to the entry of FBP1 into the nucleus. Phosphorylated FBP1 interacts with PPARα in the nucleus and translocates to the promoter region of the PPARα-mediated β-oxidation gene, where FBP1 binds to histone H3. Molecular dynamics simulations indicate that S170 phosphorylation of FBP1 alters its catalytic structural domain, allowing phosphorylated histone H3 T11 to bind tightly to FBP1 C129. Moreover, FBP1 acts as a protein phosphatase to dephosphorylate histone H3 pT11, thereby inhibiting PPARα-mediated β-oxidation gene expression and promoting energy stress-induced apoptosis. FBP1-mediated dephosphorylation of histone H3 pT11 inhibits the PPARα-mediated β-oxidation of gene transcription, leading to energetic stress-related death. In HCC tissues with higher OGT expression, FBP1 S124 undergoes O-GlcNAcylation, which blocks PERK binding to FBP1 and FBP1 S170 phosphorylation. This eliminates FBP1's inhibitory action on PPARα, resulting to accelerated β-oxidation. Increased energy generation in the mouse model supported tumor proliferation and liver tumor development [57].

It was found that FBP1 C129, R244 and D128 could form a pocket that interacted with the phosphate group of phosphorylated TERT S227 peptide, transferring the phosphoryl group of pS227 of TERT to FBP C129 while releasing dephosphorylated TERT, which could not be translocated to the nucleus, thus decreasing nuclear levels of TERT, telomerase activity, and telomere length, and promoting cancer cell senescence [58]. Moreover, the lipid nanoparticle (LNP)-mediated WT FBP1 mRNA delivery designed by this group can effectively inhibit tumor growth, finding a potential therapy for ccRCC and HCC.

FBP1 directly binds to and dephosphorylates IκBα under the effect of TNFα, thereby preventing IκBα breakdown and attenuating the NF-κB activation [59]. On the one hand inactivated NF-κB diminishes cell survival in the inflammatory microenvironment by decreasing survival genes express. On the other hand inactivated NF-κB down-regulates the expression of Tgfb1, Csf1, Csf3 and Kitl, promoting the invasion of CTLs and anti-tumor immunity via limiting MDSC mobilization.

#### FBP1 functions as a non-protein phosphatase to regulate cancer progression. Table 3

**Table 3:** FBP1 functions as a non-protein phosphatase to regulate cancer progression

Cancer types	Molecular mechanisms	Function	References
Hepatocellular carcinoma	LOXL2/Snail/FBP1/HIF-1α/VEGF	Delay the EMT process	[61, 69]
	FBP1/EZH2/ PKLR	Enhance tumor immune response	[35]
	FBP1/HMGB1	Regulate the tumor microenvironment	[72]
Pancreatic cancer	FBP1/ BRD4	Inhibit cell proliferation and metastasis	[62, 63]
Bile duct carcinoma	NFATC2/NEDD4/FBP1	Inhibit cell proliferation, migration, invasion	[19]
	FBP1/ Wnt/β-catenin	Inhibit cell proliferation, migration, invasion	[64]
	MT1JP/miR-18a-5p/FBP1	Induces apoptosis	[65]
Lung cancer	ZEB1 / FBP1	Inhibit cell growth and invasion	[21]
	FBP1/Slug	Slow down the EMT process	[70]
	LINC01419/EZH2/FBP1	Inhibit cell proliferation and stemness	[75]
Ovarian cancer	C-MYC /FBP1/STAT3	Inhibit cell proliferation, metastasis, and induces apoptosis	[22]
Esophageal squamous cell carcinoma	miR-18b-5p/ FBP1	Inhibit tumor proliferation, migration and invasion	[20]
Non small cell lung cancer	FBP1/FBXW7/NICD1	Inhibit tumor stemness	[73, 74]

In addition to promoting cellular glycolysis, HIF-1α increases the transcription of genes associated with growth and angiogenesis [60]. HIF-1α targets VEGF, a key angiogenic factor in tumor progression. Fan et al. found that LOXL2 activates the HIF-1α/VEGF pathway via the Snail-FBP1 axis, which promotes the proliferation and migration of HCC [61]. Overexpression of FBP1 may directly inhibit cancer progress and migration in breast cancer [55] and renal cell carcinoma [56] by interacting with HIF structure.

The bromodomain and extra-terminal structural domain (BET) family of proteins, including BRD2, BRD3, and BRD4, have been reported to regulate gene expression at the transcriptional level and to promote PDAC progression [62]. FBP1 downregulates the levels of the BRD4 target genes WNT5a, FLRT2, and PDE9A, and partially inhibits pancreatic cancer through BRD4 progression [63]. In cholangiocarcinoma, NFATC2 can be enriched in the promoter region of the developmentally downregulated protein 4 (NEDD4) expressed in neural precursor cells to promote its expression. In addition, NEDD4 targets FBP1 and inhibits its expression through ubiquitination, thereby promoting cholangiocarcinoma progression [19]. Overexpression of FBP1 also induces the inactivation of the Wnt/β-catenin pathway to hinder cholangiocarcinoma cell proliferation, migration, invasion, and tumorigenesis [64]. MT1JP attenuates the proliferation, migration, and invasion of cholangiocarcinoma cells and induces apoptosis by regulating the miR-18a-5p/FBP1 axis [65]. The binding of ZEB1 to the FBP1 promoter was reported to increases DNA methylation in lung cancer cells. DNA methylation in the promoter region helps reduce FBP1 expression in lung cancer cells, and in turn, the downregulation of FBP1 leads to lung cancer cell growth and invasion [21]. C-MYC is often increased in malignant tumors and has an essential function to increase cell proliferation [66]. C-MYC interacts with the FBP1 promoter and represses FBP1 transcription. STAT3 has been reported to be a potential target of FBP1. Li et al. found that not only can it bind to the trans-activating structural domain of STAT3, but FBP1 can also bind to structural domains containing the nuclear translocation signal of STAT3, retaining STAT3 in the cytoplasm, thereby blocking the binding of STAT3 to promoters of genes mediated by STAT3 and exerting an inhibitory effect on cell proliferation, metastasis, and chemoresistance [22].

Epithelial mesenchymal transition (EMT) is a biological process that allows epithelial cells to be transformed into cells with mesenchymal characteristics to gain migratory and invasive capabilities [67]. EMT promotes tumor growth and chemotherapy resistance. As a essential regulator of EMT, Snail levels is associated with cancer metastasis and reduced survival [68]. Liu et al. discovered that FBP1 plays a negative regulatory role in HCC cancer progression by inhibiting Snail-induced EMT [69]. FBP1 knockdown promotes the EMT, upregulates Slug expression, and enhances LUAD cell invasion, metastasis, and proliferation [70]. In addition, FBP1 inhibition facilitates EMT, which increases the metastasis of gastric cancer and may be a prognostic and therapeutic target [14].

HCC development is associated with metabolic and immune remodeling in the TME. NK cells are crucial immune cells in the TIME. An FBP1-deficient liver exhibits reduced numbers of NK cells and accelerated tumorigenesis. EV is an important communication carrier in the TME, delivering various biomolecules between the tumor cells and immune cells [71]. EV-delivered PKLR catalyzes phosphoenolpyruvate to pyruvate as the rate-limiting step in glycolysis. FBP1 interacts with to and decreases methyltransferase activity in the multi-comb motif protein EZH2, which induces the trimethylation of histone H3 lysine 27 and inhibits the transcription of the target gene PKLR. Thus, in hepatocytes lacking FBP1, PKLR expression is inhibited. The low PKLR level of EV, originating from FBP1-deficient hepatocytes, mainly promotes hepatic tumorigenesis by targeting NK cells, reducing the tumor immune response [35]. The hepatic TME includes fibrosis, which is mainly caused by the activation and transdifferentiation of quiescent hepatic stellate cells (HSCs) and is responsible for more than 80% of HCC cases. FBP1 lacking hepatocytes stimulate HSC activation and senescence through the release of HMGB1, which exhibits senescence-associated secretory phenotypes (SASPs), and specific growth-promoting HSC SASP components may directly contribute to liver cancer [72].

The deletion of FBP1 enhances expression of fatty acid metabolism-associated FASN, ACC1, and SREBP1C. He et al. showed that miR-18b-5p affects fatty acid metabolism and promotes ESCC proliferation, migration, and invasion by targeting FBP1 in ESCC cells [20].

The presence of cancer stem cells is thought to be the underlying reason of the difficult healing and high recurrence of various tumors. The Notch1 signaling pathway is widely believed to be related to stemness regulation, and the transmembrane receptor Notch1 can be activated by ligands expressed in neighboring cells. Through sequential cleavage, the Notch intracellular domain (NICD) is released into the cytoplasm and translocates to the nucleus, thereby activating the transcription of downstream target genes [73]. Therefore, NICD is a essential molecule for Notch signaling’s activation. He [74] et al. found that FBP1 binds to NICD1 and enhances the interaction of the E3-ubiquitin ligase FBXW7 with NICD1, decreasing the level of NICD1 through the ubiquitin–proteasome pathway. Therefore, the downregulation of FBP1 in NSCLC may lead to the overactivation of Notch signaling, thereby enhancing the cancer stem cell phenotype. Thus, targeting the FBP1/FBXW7/NICD1 axis may reduce the recurrence rate and drug resistance of NSCLC. Besides, it was demonstrated that LINC01419 could reduce FBP1 levels by recruiting EZH2, thereby promoting the proliferation and stemness of lung adenocarcinoma (LUAD) cells [75].

## Oncogenic signaling pathways involved in FBP1

Although FBP1 mainly functions as an anti-oncogene, it can also act as an oncogene to promote tumor progression. Studies have shown that FBP1 can act as an oncogene that promotes the pentose phosphate pathway, which is critical in the human body. NADPH, a scavenger of reactive oxygen species and a cofactor necessary for fatty acid synthesis, is mainly derived from the pentose phosphate pathway. Cells also produce pentose via the pentose phosphate pathway, generating ribose-5-phosphate, and leading to increased nucleotide synthesis, which is necessary for cells to divide quickly to maintain basic physiological activities. In Evi1-overexpressing cells, Evi1 binds to Fbp1 and upregulates FBP1 transcription. The transcriptional upregulation of Fbp1 and the catalase-activated pentose phosphate pathway are critical for the progression of Evi1-driven leukemias. On the other hand, the inhibition of FBP1 does not impair normal hematopoiesis. Thus, targeting FBP1 in highly Evi1-overexpressing leukemias may be an ideal therapeutic approach [76].

## Regulation of transcriptional levels and post-transcriptional modifications of FBP1 Fig. 2

**Fig. 2:**
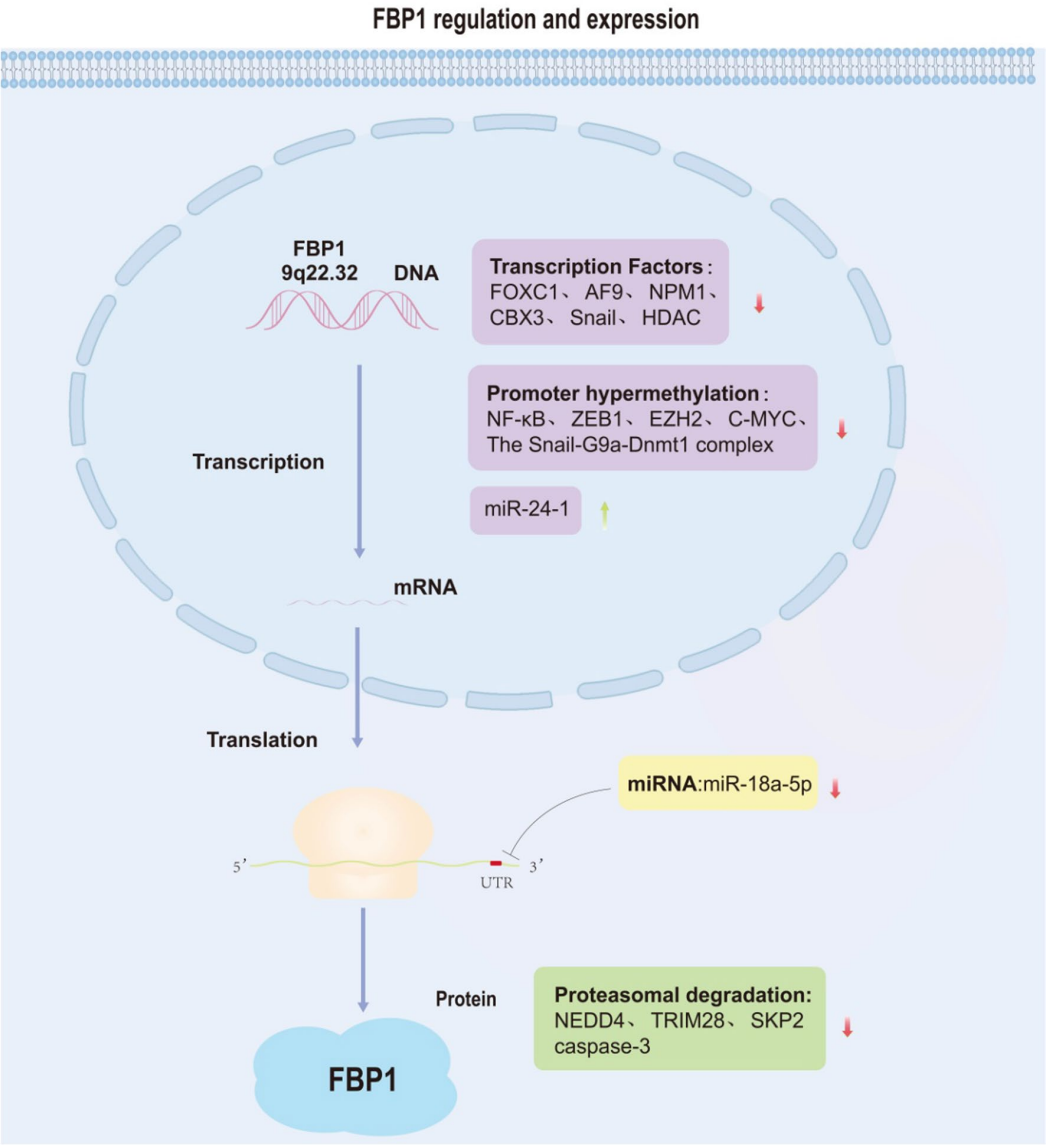
Regulation of transcriptional levels and post-transcriptional modifications of FBP1

FBP1 expression can occur at the transcriptional, translational, or degradative level. Several transcription factors interact with the promoter of FBP1 and repress transcription. These transcription factors include FOXC1, AF9, NPM1, CBX3, and Snail. In colorectal cancer, FOXC1 directly binds to the promoter region of the FBP1 gene and negatively regulates its transcriptional activity [43], whereas, AF9 reduces fbp1 transcription by targeting the FBP1 promoter through recognition of H3K9ac [50]. In HCC, HDAC inhibits FBP1 transcription by reducing histone H3K27Ac in the FBP1 enhancer region [47], whereas, NPM1 directly binds to the FBP1 promoter region at the E-box motif and reduces FBP1 transcription [15]. CBX3 occupies the promoter region of FBP1 and represses the activity of the FBP1 promoter [41]. Hypermethylation of the FBP1 promoter region is the primary cause behind FBP1 deletion in many cancers [77]. Chen et al. discovered that promoter hypermethylation usually lowers FBP1 in the majority of hepatocellular and colon cancer cell lines as well as primary tumor tissues. Promoter hypermethylation-mediated FBP1 silencing can be restored by pharmacological demethylation [67]. In gastric cancer [44], glioma [45], and LUAD cells [46], NF-κB increases the promoter methylation of the FBP1 gene, which decreases the expression of the FBP1 protein. Thus, a novel biomarker for the prognosis of gastric cancer may be the promoter methylation of FBP1. In basal-like breast cancer cells, the Snail-G9a-Dnmt1 complex is essential for silencing the E-calmodulin promoter and is also the cause of methylation of FBP1 [78]. Additionally, ZEB1 increases DNA methylation in lung cancer cells67 via binding to the FBP1 promoter [21]. EZH2, a core component of PRC2 induces H3K27 trimethylation (H3K27me3) to repress FBP1 expression [48]. miR-24-1 activates FBP1 at the transcriptional level with enhancer activity [51]. In ovarian cancer, C-MYC binds to the promoter region of FBP1 and promotes its methylation [22]. At the translational level, miR-18a-5p binds to the FBP1 3′ untranslated region to inhibit FBP1 expression [65]. In HCC, the copy number loss of FBP1 can also lead to decreased FBP1 expression [79]. In addition, the level of FBP1 expression depends on the degradation system. In prostate cancer (PCa), deletion of PTEN activates the PI3K/AKT pathway to promote FBP1 protein degradation through two mechanisms. Activated AKT promotes CDK-dependent phosphorylation of FBP1 by downregulating p27, which increases the activity of its substrate proteins, including CDKs. In addition, activated AKT increases SKP2 expression at the transcriptional level, thereby ubiquitinating the degradation of phosphorylated FBP1 [18]. Studies have shown that activation of the PI3K/AKT/mTOR pathway enhances FBP stability by inactivating caspase-3/7 [80]. Therefore, targeting the PI3K/AKT/mTOR pathway may be an effective treatment for HCC patients with high FBP expression levels. NEDD4 promotes cholangiocarcinoma progression by targeting FBP1 and inhibiting its expression through ubiquitination [19]. H254R-mutant FBP1 enhances its ubiquitination and proteasomal degradation [81]. TRIM28 acts as an E3 ubiquitin ligase for FBP1 in HCC and inhibits the expression of FBP1 via ubiquitination. JQ1, as one of the inhibitors of the BET structural domain, increases the expression of FBP1 by interfering with the interaction between FBP1 and TRIM28 [49]. By binding to FBP1 and causing FBP1 to become deubiquitinated, USP7 stops FBP1 from moving into the nucleus. In PDAC patients, USP7 inhibitors combined with PARP inhibitors could produce stronger effects than PARP inhibitors alone [82].

In addition to methylation and ubiquitination, which are essential for regulating FBP1 expression, other post-translational modifications play important roles in cancer biometabolism. Tip60 and HDAC3 regulate the diacetylation of FBP1 in pancreatic cancer cells. The diacetylation of FBP1 at the Thr110 and Thr113 sites is crucial for FBP1-BRD4 interaction [63]. When PTEN is lost, FBP1 is phosphorylated at the CDK site, which increases its degradation [18].

## FBP1 is involved in drug resistance in tumors

The emergence of drug resistance in tumor therapy is a major challenge. The cause of drug resistance is multifaceted as various factors, such as enhanced cellular resistance to apoptosis, improved DNA damage repair, and the upregulated expression of ATP-dependent drug transporter proteins, may be involved. Abundant clinical and experimental evidence suggests that low levels of intracellular FBP1 are associated with drug resistance in tumor cells.

### FBP1 enhances tumor sensitivity to radiation and chemotherapy by inhibiting glycolysis

Compared to normal nasopharyngeal epithelial cells, nasopharyngeal cancer cells exhibit decreased levels of FBP1 expression. FBP1 silencing lessens radiosensitivity by promoting glycolysis and hampers radiation-induced apoptosis and DNA damage. A mechanistic exploration revealed that FBP1 increases FBXW7 protein levels by inhibiting the auto-ubiquitination of FBXW7, which promotes mTOR ubiquitination to inhibit mTOR levels, thereby inhibiting glycolysis and enhancing the anti-tumor effects of radiation [83]. Higher levels of FBP1 increases susceptibility to cisplatin [84]. Cisplatin inhibits glycolysis in tumor cells [85]. Inhibiting glycolysis has been demonstrated to improve drug-induced apoptosis in patients with ovarian cancer, lung cancer, and leukemia [86]. Thus, inhibiting glycolysis by increasing FBP1 expression may play a crucial role in improving cisplatin sensitivity, and combination therapies targeting the glycolytic pathway are promising for the treatment of chemoresistant cancer cells.

### FBP1 inhibition can ameliorate chemotherapy resistance

FBP1 inhibition ameliorates chemotherapy resistance in Evi1 upregulated leukemia patients by reducing oxidative phosphorylation (OXPHOS) [76]. Some studies have shown that AML with high OXPHOS activity is associated with chemotherapy resistance [87]. Evi1 overexpression upregulates OXPHOS with Evi1-high AML, resulting in formidable resistance to conventional chemotherapy in the clinical setting. In contrast to Evi1 overexpression, FBP1 overexpression alone partially induces OXPHOS activation. This OXPHOS upregulation may be indirectly induced by ATP deficiency associated with glycolytic downregulation. Together, these findings imply that inhibiting FBP1 may increase chemotherapy sensitivity in individuals with Evi1-high leukemia by reducing OXPHOS [87].

### FBP1 regulates the sensitivity of target therapy

BET inhibitors show antitumor activity in KRAS-driven cancers [88]. A well-studied BET protein-selective inhibitor that has been demonstrated to prevent pancreatic cancer development by inhibiting the expression of c-MYC, FOSL, and HMGA2 [89]. However, pancreatic cancer cells typically acquire resistance to BET inhibitors [90] and c-MYC is thought to be responsible for this [91]. The c-MYC protein is an oncogenic transcription factor that regulates at least 15% of proliferation, differentiation, and metabolism-related genes in pancreatic cancer cells [92]. The KRAS/ERK/c-MYC axis is a major driver of pancreatic cancer tumorigenesis [93]. FBP1 has been discovered as a new molecule that modulates the sensitivity of BET inhibitors. According to studies, JQ1 disturbs the link between TRIM28 and FBP1, improving FBP1 protein levels, and FBP1 increases c-Myc degradation by blocking the MAPK pathway, making pancreatic cancer more sensitive to JQ1 [94]. Gemcitabine plays an important role in PDAC treatment. Gemcitabine resistance is due to ERK activation [95]. FBP1 increases the sensitivity of pancreatic cancer to gemcitabine by binding to IQGAP1, impeding IQGAP1-dependent ERK1/2 phosphorylation and activation. Thus, blocking ERK activation by treating the cells with an FBP1-derived small-peptide inhibitor is effective in treating PDAC [96]. Nuclear FBP1 regulates pancreatic cancer sensitivity to PARP drugs by binding to DNMT1 and trapping PARP1 in chromatin [82].

β-Elemene induced apoptosis in gefitinib-resistant lung cancer cells through the FBP1/STAT3 axis, which inhibited cancer cell growth and increasing sensitivity to gefitinib. Thus, FBP1 may be an effective target for treating gefitinib-resistant patients [97].

Reduced FBP1 expression was reported to promote tumor growth and resistance to immune checkpoint blockade therapy in mice [11]. Because FBP1 deletion contributes to PD-L1 upregulation and resistance to anti-PD-L1 therapy, FBP1 deletion may be associated with immune evasion in human cancers.

## Chemical inhibitors for restoring FBP1 expression

### Chemical inhibitors that restore FBP1 expression are mainly categorized into four groups

Small molecules targeting promoter methylation (e.g., 5AZA). DNA methylation inhibitors have achieved significant efficacy in cancer treatment. 5AZA, a DNA methyltransferase (DNMT) inhibitor that kills tumor cells by reactivating genes that have been silenced by methylation, has been approved by the FDA for the treatment of myelodysplastic syndromes. Since 5AZA can reactivate the immune response of tumors, DNA methylation inhibitors combined with immunotherapy have been applied to cancer treatment with remarkable results [98], but the specific mechanism remains unelucidated. Studies have shown that the treatment of cells with 5AZA can substantially increase the expression of FBPase mRNA in breast, gastric, and lung cancers [21].

### HDAC inhibitors targeting promoter acetylation

Studies have shown that HDAC inhibitors restore FBP1 expression by mediating the reduction of H3K27Ac in the FBP1 enhancer, reduce glucose lowering and lactate secretion, and inhibit HCC cell growth and tumor growth in mice in vitro [47, 99], and it has been demonstrated that HDAC inhibitors such as tricotinostatin A (TSA), sodium butyrate (NaBu) [100], SAHA [101], and LBH589 [102],which can exert better effects in combination with 5AZA. HDAC inhibitors that have been used in the clinic are vorinostat (SAHA), belinostat (PXD101), pabilostat (LBH589), and romidepsin (FK228), cidabenamide (chidamide, CS055). There are also several HDACi in clinical trials. However, numerous animal and clinical studies are needed to confirm the specific efficacy of HDAC inhibitors in cancers with low FBP1 expression.

### Small molecule that inhibits ubiquitinated degradation of FBP1

It has been demonstrated that bortezomib suppresses the apoptotic activity of HCC by inhibiting FBP1 degradation and modulating the Warburg effect in HCC, thus inhibiting HCC proliferation, migration, and invasion, but further clinical trials are needed to prove its value in clinical application [103]. JQ1 reduces the ubiquitination of FBP1 by disrupting the interaction between FBP1 and TRIM28, suggesting that JQ1 may be an ideal small molecule to increase FBP1 expression and inhibit pancreatic cancer [49].

### Other drugs

Drugs targeting regulators upstream of FBPase, such as BAY11-7085, which inhibits NF-ΚB activity, and IkB-α M. Unfortunately there are currently no drugs that can directly target FBP1 to restore its expression. Although these chemical inhibitors have been shown to restore FBP1 expression in tumors, thereby inhibiting tumor progression, and some of them have been used clinically. However, these small molecule inhibitors do not act by directly targeting FBP1, and studies of their effects on FBP1 expression remain at the preclinical study stage.Therefore, it is crucial to conduct preclinical and clinical studies on these drugs in patients with low FBP1-expressing cancers and to develop new small molecule inhibitors that directly target FBP1.

## Pyruvate kinase M2 and its role in cancer

Pyruvate kinase is an enzyme that catalyzes the conversion of phosphoenolpyruvate and ADP to pyruvate and ATP in glycolysis and plays a role in the regulation of cellular metabolism. Pyruvate kinase has four isoforms that shift from the adult M1 isoform to the embryonic M2 isoform (PKM2) in tumor tissue [104]. In contrast to the function of FBP1 in inhibiting glycolysis, PKM2, a key enzyme in promoting glycolysis, was significantly upregulated in tumor tissues [105, 106]. Elevated expression of PKM2 has been reported in hepatocellular carcinoma [107], cholangiocarcinoma [108] and other tumors, and the driver genes of adrenocortical carcinoma, PRKAR-1A, CTNNB1, ZNRF3, TP53, CCNE1, and TERF2, were closely related to PKM2 [109]. PKM2 are required for aerobic glycolysis, which provides a selective growth advantage for tumor cells in vivo. Conversion between the tetrameric and dimeric forms of PKM2 allows tumor cells to survive in environments with varying oxygen and nutrient supplies [110]. In addition to being a key enzyme in glycolysis, PKM2 also possesses protein kinase activity [111]. Phosphorylated targets identified include histone H3 T11 [112], STAT3 Y705 [113] BUB3 Y207 [114] and MLC2 Y118 [115]. PKM2 regulates gene expression or the cell cycle through nonclassical pyruvate kinase function.

(PKM2) has emerged as a potential target for anticancer therapy [116]. Various antitumor agents have been developed against PKM2 targets [117]. Jyotika Jadhav et al. developed a series of ferrocene-based tumor PKM2 modulators, and some of these compounds showed high efficacy and specificity on CAL27 cells, a representative of human oral squamous cell carcinoma (OSCC) [118]. PKM2 inhibitors based on thiazole design show unique efficacy in the treatment of triple-negative breast cancer [119]. Novel sulfonamide derivative 9b compounds can exert good antitumor effects in lung cancer [120]; in the designed novel tetrazole-based derivatives, 1-(imidazo[1,2-a]pyrimidin-3-yl)−2-(5-(naphthalen-2-yl)−2H- tetrazol-2-yl)ethan-1-one (9b) exhibits potent and selective antiproliferative activity against U87MG glioma cells and is expected to be a therapeutic candidate against glioblastoma (GB) [121]. Although several PKM2 modulators have been developed, extensive preclinical and clinical studies are needed to explore the clinical application of these modulators and whether their combination with FBP1 modulators has a unique efficacy in tumor treatment.

## Future perspectives

Metabolic alterations are the essence of tumorigenesis [122, 123]. Increasing evidence suggests that aerobic glycolysis plays a key role in tumorigenesis and cancer progression [124]. FBP1 plays a negative regulatory role in cancer progression by antagonizing glycolysis, inhibiting epithelial-mesenchymal transition (EMT), thus promoting apoptosis and other biological processes [125]. In addition to being an anti-oncogene, FBP1 was found to act as an oncogene promoting the pentose phosphate pathway in Evi1-overexpressing leukemia cells [76]. The biological function of FBP1 in tumors is complex and requires further studies for a better understanding.

FBP1 deficiency is prevalent in cancer and is associated with poor prognosis [126]. Although many studies have demonstrated that FBP1 can be used as a potential tumor biomarker to guide clinical diagnosis and treatment [127, 128], it is not currently used in the clinic. On the one hand, the expression pattern of FBP1 is complex.FBP1 is not only involved in tumor progression, but also plays a role in other diseases, such as metabolic disorders and related diseases. Its potential application as a biomarker requires more research and validation; on the other hand, the detection technology of FBP1 is not mature enough to be widely used in clinical settings. To solve this problem, researchers need to conduct large-scale clinical studies and accumulate more clinical data to prove the validity and reliability of FBP1 as a tumor biomarker. Meta-analysis can also be used to combine data to increase the sample size and the credibility of the conclusions. Moreover, the development and optimization of FBP1 detection technology can improve the sensitivity and specificity of the assay, reduce the cost of the assay, and make it easier to promote its application in the clinic. In addition, interdisciplinary collaboration by integrating resources from biology, medicine, engineering and other fields can also help the research and application of FBP1 as a biomarker.

Although a large number of studies have shown that targeting FBP1 may be an effective tumor therapy [129], there are still many limitations and challenges in targeting FBP1. First, due to the complex biological functions of FBP1 such as glucose metabolism, cell proliferation, and apoptosis, it is a challenge to design specific drugs that can effectively inhibit the activity of FBP1 without affecting the functions of other similar enzymes. In addition, due to the major function of FBP1 in cellular metabolism, any intervention on it may bring unpredictable side effects. For example, inhibition of FBP1 activity may lead to disruption of glucose metabolism, which in turn affects cellular energy supply and growth cycle. Studies have shown that FBP1 expression and activity may be regulated by multiple molecules, including FBP2 (fructose-1,6-bisphosphatase 2) and other related metabolic enzymes. Therefore, targeting FBP1 may require simultaneous consideration of its interactions with other metabolic pathways.

FBP1 is recognized as a drug resistance-associated protein in cancer [130]. Low expression of the FBP1 protein may promote drug resistance through complex molecular mechanisms. Therefore, predicting patient sensitivity to therapy by testing FBP1 expression would benefit patients who are unlikely to respond to drugs and would protect patients from unnecessary drug side effects. Moreover,restoring FBP1 expression has the potential to become a new strategy to overcome drug resistance. Overall, FBP1 is valuable for the diagnosis, treatment, and prognosis of various cancers. Hence, the development of therapeutic drugs targeting FBP1 is of interest.

## Data Availability

No datasets were generated or analysed during the current study. No, I do not have any research data outside the submitted manuscript file.

## References

[1] Raafat El-Maghrabi M, Lange AJ, Jiang W, et al. Human fructose-1,6-bisphosphatase gene (FBP1): exon-intron organization, localization to chromosome bands 9q22.2-q22.3, and mutation screening in subjects with fructose-1,6-bisphosphatase deficiency. Genomics. 1995;27(3):520–5. 7558035 10.1006/geno.1995.1085

[2] Benkovic SJ, deMaine MM. Mechanism of action of fructose 1,6-bisphosphatase. Adv Enzymol Relat Areas Mol Biol. 1982;53:45–82. 6277165 10.1002/9780470122983.ch2

[3] Kaur R, Dahiya L, Kumar M. Fructose-1, 6-bisphosphatase inhibitors: a new valid approach for management of type 2 diabetes mellitus[J]. Eur J Med Chem. 2017;141:473–505. 29055870 10.1016/j.ejmech.2017.09.029

[4] Moreno-Sánchez R, Rodríguez-Enríquez S, Marín-Hernández A, et al. Energy metabolism in tumor cells. FEBS J. 2007;274(6):1393–418. 17302740 10.1111/j.1742-4658.2007.05686.x

[5] Kim JW, Dang CV. Cancer’s molecular sweet tooth and the Warburg effect. Cancer Res. 2006;66(18):8927–30. 16982728 10.1158/0008-5472.CAN-06-1501

[6] Warburg O, Wind F, Negelein E. THE METABOLISM OF TUMORS IN THE BODY. J Gen Physiol. 1927;8(6):519–30. 19872213 10.1085/jgp.8.6.519 PMC2140820

[7] Duncan R, Bazar L, Michelotti G, et al. A sequence-specific, single-strand binding protein activates the far upstream element of c-myc and defines a new DNA-binding motif. Genes Dev. 1994;8(4):465–80. 8125259 10.1101/gad.8.4.465

[8] Chien HL, Liao CL, Lin YL. FUSE binding protein 1 interacts with untranslated regions of Japanese encephalitis virus RNA and negatively regulates viral replication. J Virol. 2011;85(10):4698–706. 21367899 10.1128/JVI.01950-10 PMC3126168

[9] Chung HJ, Liu J, Dundr M, et al. FBPs are calibrated molecular tools to adjust gene expression. Mol Cell Biol. 2006;26(17):6584–97. 16914741 10.1128/MCB.00754-06 PMC1592819

[10] Han F, Guo S, Huang C, et al. Gastric cancer mesenchymal stem cells inhibit natural killer cell function by up-regulating FBP1. Cent Eur J Immunol. 2021;46(4):427–37. 35125940 10.5114/ceji.2021.111753 PMC8808309

[11] Wang B, Zhou Y, Zhang J, et al. Fructose-1,6-bisphosphatase loss modulates STAT3-dependent expression of PD-L1 and cancer immunity. Theranostics. 2020;10(3):1033–45. 31938049 10.7150/thno.38137 PMC6956820

[12] Du D, Liu C, Qin M, et al. Metabolic dysregulation and emerging therapeutical targets for hepatocellular carcinoma. Acta Pharm Sin B. 2022;12(2):558–80. 35256934 10.1016/j.apsb.2021.09.019 PMC8897153

[13] Li B, Qiu B, Lee DS, et al. Fructose-1,6-bisphosphatase opposes renal carcinoma progression. Nature. 2014;513(7517):251–5. 25043030 10.1038/nature13557 PMC4162811

[14] Li J, Wang Y, Li QG, et al. Downregulation of FBP1 Promotes Tumor Metastasis and Indicates Poor Prognosis in Gastric Cancer via Regulating Epithelial-Mesenchymal Transition. PLoS ONE. 2016;11(12): e0167857. 27978536 10.1371/journal.pone.0167857 PMC5158319

[15] Zhu Y, Shi M, Chen H, et al. NPM1 activates metabolic changes by inhibiting FBP1 while promoting the tumorigenicity of pancreatic cancer cells. Oncotarget. 2015;6(25):21443–51. 26068981 10.18632/oncotarget.4167 PMC4673277

[16] Li Q, Wei P, Wu J, et al. The FOXC1/FBP1 signaling axis promotes colorectal cancer proliferation by enhancing the Warburg effect. Oncogene. 2019;38(4):483–96. 30171256 10.1038/s41388-018-0469-8

[17] Fu D, Li J, Wei J, et al. HMGB2 is associated with malignancy and regulates Warburg effect by targeting LDHB and FBP1 in breast cancer. Cell Commun Signal. 2018;16(1):8. 29463261 10.1186/s12964-018-0219-0 PMC5819211

[18] Song C, Zhang J, Liu X, et al. PTEN loss promotes Warburg effect and prostate cancer cell growth by inducing FBP1 degradation. Front Oncol. 2022;27(12): 911466. 10.3389/fonc.2022.911466 PMC9552847 36237339

[19] Zhao W, Zhao J, Li K, et al. Oncogenic Role of the NFATC2/NEDD4/FBP1 Axis in Cholangiocarcinoma. Lab Invest. 2023;103(9): 100193. 37285922 10.1016/j.labinv.2023.100193

[20] He Y, Hua R, Li B, et al. Loss of FBP1 promotes proliferation, migration, and invasion by regulating fatty acid metabolism in esophageal squamous cell carcinoma. Aging (Albany NY). 2020;13(4):4986–98. 33232284 10.18632/aging.103916 PMC7950246

[21] Zhang J, Wang J, Xing H, et al. Down-regulation of FBP1 by ZEB1-mediated repression confers to growth and invasion in lung cancer cells. Mol Cell Biochem. 2016;411(1–2):331–40. 26546081 10.1007/s11010-015-2595-8

[22] Li H, Qi Z, Niu Y, et al. FBP1 regulates proliferation, metastasis, and chemoresistance by participating in C-MYC/STAT3 signaling axis in ovarian cancer. Oncogene. 2021;40(40):5938–49. 34363022 10.1038/s41388-021-01957-5 PMC8497274

[23] Li B. Recent proceedings in research of tumor-associated disorders of glucose metabolism[J]. J Sun Yat-sen Univ (Med Sci). 2017;2:222–8.

[24] Yip J, Geng X, Shen J, et al. Cerebral gluconeogenesis and diseases[J]. Front Pharmacol. 2016;7:521. 28101056 10.3389/fphar.2016.00521 PMC5209353

[25] Pilkis SJ, Claus TH. Hepatic gluconeogenesis/glycolysis: regulation and structure/function relationships of substrate cycle enzymes[J]. Annu Rev Nutr. 1991;11:465–515. 1892710 10.1146/annurev.nu.11.070191.002341

[26] He L, Weber A, Levens D. Nuclear targeting determinants of the far upstream element binding protein, a c-myc transcription factor. Nucleic Acids Res. 2000;28(22):4558–65. 11071946 10.1093/nar/28.22.4558 PMC113884

[27] Michelotti GA, Michelotti EF, Pullner A, et al. Multiple single-stranded cis elements are associated with activated chromatin of the human c-myc gene in vivo. Mol Cell Biol. 1996;16(6):2656–69. 8649373 10.1128/mcb.16.6.2656 PMC231256

[28] Holstege FC, Fiedler U, Timmers HT. Three transitions in the RNA polymerase II transcription complex during initiation. EMBO J. 1997;16(24):7468–80. 9405375 10.1093/emboj/16.24.7468 PMC1170346

[29] Duncan R, Collins I, Tomonaga T, et al. A unique transactivation sequence motif is found in the carboxyl-terminal domain of the single-strand-binding protein FBP. Mol Cell Biol. 1996;16(5):2274–82. 8628294 10.1128/mcb.16.5.2274 PMC231215

[30] Valverde R, Edwards L, Regan L. Structure and function of KH domains. FEBS J. 2008;275(11):2712–26. 18422648 10.1111/j.1742-4658.2008.06411.x

[31] Olanich ME, Moss BL, Piwnica-Worms D, et al. Identification of FUSE-binding protein 1 as a regulatory mRNA-binding protein that represses nucleophosmin translation. Oncogene. 2011;30(1):77–86. 20802533 10.1038/onc.2010.404 PMC3190308

[32] Zheng Y, Miskimins WK. Far upstream element binding protein 1 activates translation of p27Kip1 mRNA through its internal ribosomal entry site. Int J Biochem Cell Biol. 2011;43(11):1641–8. 21855647 10.1016/j.biocel.2011.08.001 PMC3206725

[33] Shi L, Huang H, Lu X, et al. Effect of human umbilical cord-derived mesenchymal stem cells on lung damage in severe COVID-19 patients: a randomized, double-blind, placebo-controlled phase 2 trial. Signal Transduct Target Ther. 2021;6(1):58. 33568628 10.1038/s41392-021-00488-5 PMC7873662

[34] Cong J, Wang X, Zheng X, et al. Dysfunction of Natural Killer Cells by FBP1-Induced Inhibition of Glycolysis during Lung Cancer Progression. Cell Metab. 2018;28(2):243-255.e5. 30033198 10.1016/j.cmet.2018.06.021

[35] Liu Z, You Y, Chen Q, et al. Extracellular vesicle-mediated communication between hepatocytes and natural killer cells promotes hepatocellular tumorigenesis. Mol Ther. 2022;30(2):606–20. 34601133 10.1016/j.ymthe.2021.07.015 PMC8821954

[36] Li P, Li K, Yuan W, et al. 1α,25(OH)_2_D_3_ ameliorates insulin resistance by alleviating γδ T cell inflammation via enhancing fructose-1,6-bisphosphatase 1 expression. Theranostics. 2023;13(15):5290–304. 37908738 10.7150/thno.84645 PMC10614678

[37] Seuter S, Pehkonen P, Heikkinen S, et al. Dynamics of 1α,25-dihydroxyvitamin D3-dependent chromatin accessibility of early vitamin D receptor target genes. Biochim Biophys Acta. 2013;1829(12):1266–75. 24185200 10.1016/j.bbagrm.2013.10.003

[38] Guo Q, Li K, Jiang N, et al. A novel risk model of three gefitinib-related genes FBP1, SBK1 and AURKA is related to the immune microenvironment and is predicting prognosis of lung adenocarcinoma patients. Aging (Albany NY). 2023;15(18):9633–60. 37737707 10.18632/aging.205040 PMC10564433

[39] Yang J, Wang C, Zhao F, et al. Loss of FBP1 facilitates aggressive features of hepatocellular carcinoma cells through the Warburg effect. Carcinogenesis. 2017;38(2):134–43. 27742690 10.1093/carcin/bgw109

[40] Chen M, Zhang J, Li N, et al. Promoter hypermethylation mediated downregulation of FBP1 in human hepatocellular carcinoma and colon cancer. PLoS ONE. 2011;6(10): e25564. 22039417 10.1371/journal.pone.0025564 PMC3198434

[41] Chen LY, Cheng CS, Qu C, et al. CBX3 promotes proliferation and regulates glycolysis via suppressing FBP1 in pancreatic cancer. Biochem Biophys Res Commun. 2018;500(3):691–7. 29678579 10.1016/j.bbrc.2018.04.137

[42] Zhang B, Li D, Jin X, et al. The CDK4/6 inhibitor PD0332991 stabilizes FBP1 by repressing MAGED1 expression in pancreatic ductal adenocarcinoma. Int J Biochem Cell Biol. 2020;128: 105859. 32987196 10.1016/j.biocel.2020.105859

[43] Yu J, Li J, Chen Y, et al. Snail Enhances Glycolysis in the Epithelial-Mesenchymal Transition Process by Targeting FBP1 in Gastric Cancer. Cell Physiol Biochem. 2017;43(1):31–8. 28848200 10.1159/000480314

[44] Liu X, Wang X, Zhang J, et al. Warburg effect revisited: an epigenetic link between glycolysis and gastric carcinogenesis. Oncogene. 2010;29(3):442–50. 19881551 10.1038/onc.2009.332

[45] Chen Z, Bao H, Long J, et al. GBE1 Promotes Glioma Progression by Enhancing Aerobic Glycolysis through Inhibition of FBP1. Cancers (Basel). 2023;15(5):1594. 36900384 10.3390/cancers15051594 PMC10000543

[46] Li L, Yang L, Fan Z, et al. Hypoxia-induced GBE1 expression promotes tumor progression through metabolic reprogramming in lung adenocarcinoma. Signal Transduct Target Ther. 2020;5(1):54. 32439898 10.1038/s41392-020-0152-8 PMC7242448

[47] Yang J, Jin X, Yan Y, et al. Inhibiting histone deacetylases suppresses glucose metabolism and hepatocellular carcinoma growth by restoring FBP1 expression. Sci Rep. 2017;6(7):43864. 10.1038/srep43864 PMC5338333 28262837

[48] Liao K, Deng S, Xu L, et al. A Feedback Circuitry between Polycomb Signaling and Fructose-1, 6-Bisphosphatase Enables Hepatic and Renal Tumorigenesis. Cancer Res. 2020;80(4):675–88. 31948940 10.1158/0008-5472.CAN-19-2060

[49] Jin X, Pan Y, Wang L, et al. MAGE-TRIM28 complex promotes the Warburg effect and hepatocellular carcinoma progression by targeting FBP1 for degradation. Oncogenesis. 2017;6(4): e312. 28394358 10.1038/oncsis.2017.21 PMC5520498

[50] He X, Zhong X, Fang Y, et al. AF9 sustains glycolysis in colorectal cancer via H3K9ac-mediated PCK2 and FBP1 transcription. Clin Transl Med. 2023;13(8): e1352. 37565737 10.1002/ctm2.1352 PMC10413954

[51] Ju D, Liang Y, Hou G, et al. FBP1 /miR-24-1/enhancer axis activation blocks renal cell carcinoma progression via Warburg effect. Front Oncol. 2022;1(12): 928373. 10.3389/fonc.2022.928373 PMC9376222 35978816

[52] Iyer NV, Kotch LE, Agani F, et al. Cellular and developmental control of O2 homeostasis by hypoxia-inducible factor 1 alpha. Genes Dev. 1998;12(2):149–62. 9436976 10.1101/gad.12.2.149 PMC316445

[53] Zhong H, De Marzo AM, Laughner E, et al. Overexpression of hypoxia-inducible factor 1alpha in common human cancers and their metastases. Cancer Res. 1999;59(22):5830–5. 10582706

[54] Perou CM, Sørlie T, Eisen MB, et al. Molecular portraits of human breast tumours. Nature. 2000;406(6797):747–52. 10963602 10.1038/35021093

[55] Shi L, He C, Li Z, et al. FBP1 modulates cell metabolism of breast cancer cells by inhibiting the expression of HIF-1α. Neoplasma. 2017;64(4):535–42. 28485159 10.4149/neo_2017_407

[56] Ning XH, Li T, Gong YQ, et al. Association between FBP1 and hypoxia-related gene expression in clear cell renal cell carcinoma. Oncol Lett. 2016;11(6):4095–8. 27313747 10.3892/ol.2016.4504 PMC4888282

[57] Wang Z, Li M, Jiang H, et al. Fructose-1,6-bisphosphatase 1 functions as a protein phosphatase to dephosphorylate histone H3 and suppresses PPARα-regulated gene transcription and tumour growth. Nat Cell Biol. 2022;24(11):1655–65. 36266488 10.1038/s41556-022-01009-4

[58] Li M, Wang Z, Tao J, et al. Fructose-1,6-bisphosphatase 1 dephosphorylates and inhibits TERT for tumor suppression. Nat Chem Biol. 2024;20(11):1547. 38538923 10.1038/s41589-024-01597-2

[59] Zhu W, Chu H, Zhang Y, et al. Fructose-1,6-bisphosphatase 1 dephosphorylates IκBα and suppresses colorectal tumorigenesis. Cell Res. 2023;33(3):245–57. 36646759 10.1038/s41422-022-00773-0 PMC9977772

[60] Carmeliet P, Dor Y, Herbert JM, et al. Role of HIF-1alpha in hypoxia-mediated apoptosis, cell proliferation and tumour angiogenesis. Nature. 1998;394(6692):485–90. 9697772 10.1038/28867

[61] Fan Z, Zheng W, Li H, et al. LOXL2 upregulates hypoxia-inducible factor-1α signaling through Snail-FBP1 axis in hepatocellular carcinoma cells. Oncol Rep. 2020;43(5):1641–9. 32323822 10.3892/or.2020.7541 PMC7107812

[62] Jang MK, Mochizuki K, Zhou M, et al. The bromodomain protein Brd4 is a positive regulatory component of P-TEFb and stimulates RNA polymerase II-dependent transcription. Mol Cell. 2005;19(4):523–34. 16109376 10.1016/j.molcel.2005.06.027

[63] Yang C, Zhu S, Yang H, et al. FBP1 binds to the bromodomain of BRD4 to inhibit pancreatic cancer progression. Am J Cancer Res. 2020;10(2):523–35. 32195024 PMC7061763

[64] Zhao W, Yang S, Chen J, et al. Forced overexpression of FBP1 inhibits proliferation and metastasis in cholangiocarcinoma cells via Wnt/β-catenin pathway. Life Sci. 2018;1(210):224–34. 10.1016/j.lfs.2018.09.009 30193944

[65] Zhao W, Zhao J, Guo X, et al. LncRNA MT1JP plays a protective role in intrahepatic cholangiocarcinoma by regulating miR-18a-5p/FBP1 axis. BMC Cancer. 2021;21(1):142. 33557774 10.1186/s12885-021-07838-0 PMC7871555

[66] Dang CV. MYC on the path to cancer. Cell. 2012;149(1):22–35. 22464321 10.1016/j.cell.2012.03.003 PMC3345192

[67] De Craene B, Berx G. Regulatory networks defining EMT during cancer initiation and progression. Nat Rev Cancer. 2013;13(2):97–110. 23344542 10.1038/nrc3447

[68] Yang MH, Chen CL, Chau GY, et al. Comprehensive analysis of the independent effect of twist and snail in promoting metastasis of hepatocellular carcinoma. Hepatology. 2009;50(5):1464–74. 19821482 10.1002/hep.23221

[69] Liu GM, Li Q, Zhang PF, et al. Restoration of FBP1 suppressed Snail-induced epithelial to mesenchymal transition in hepatocellular carcinoma. Cell Death Dis. 2018;9(11):1132. 30429463 10.1038/s41419-018-1165-x PMC6235921

[70] Wang Z, He T, Lv W, et al. Down-regulation of FBP1 in lung adenocarcinoma cells promotes proliferation and invasion through SLUG mediated epithelial mesenchymal transformation. Transl Cancer Res. 2023;12(2):236–46. 36915593 10.21037/tcr-22-2200 PMC10007873

[71] Umezu T, Tadokoro H, Azuma K, et al. Exosomal miR-135b shed from hypoxic multiple myeloma cells enhances angiogenesis by targeting factor-inhibiting HIF-1. Blood. 2014;124(25):3748–57. 25320245 10.1182/blood-2014-05-576116 PMC4263983

[72] Li F, Huangyang P, Burrows M, et al. FBP1 loss disrupts liver metabolism and promotes tumorigenesis through a hepatic stellate cell senescence secretome. Nat Cell Biol. 2020;22(6):728–39. 32367049 10.1038/s41556-020-0511-2 PMC7286794

[73] Wilson JJ, Kovall RA. Crystal structure of the CSL-Notch-Mastermind ternary complex bound to DNA. Cell. 2006;124(5):985–96. 16530045 10.1016/j.cell.2006.01.035

[74] He T, Wang Y, Lv W, et al. FBP1 inhibits NSCLC stemness by promoting ubiquitination of Notch1 intracellular domain and accelerating degradation. Cell Mol Life Sci. 2024;81(1):87. 38349431 10.1007/s00018-024-05138-x PMC10864425

[75] Chen Z, Tang W, Zhou Y, et al. The role of LINC01419 in regulating the cell stemness in lung adenocarcinoma through recruiting EZH2 and regulating FBP1 expression. Biol Direct. 2022;17(1):23. 36050791 10.1186/s13062-022-00336-8 PMC9438337

[76] Mizuno H, Koya J, Masamoto Y, et al. Evi1 upregulates Fbp1 and supports progression of acute myeloid leukemia through pentose phosphate pathway activation. Cancer Sci. 2021;112(10):4112–26. 34363719 10.1111/cas.15098 PMC8486204

[77] Bigl M, Jandrig B, Horn LC, et al. Aberrant methylation of human L- and M-fructose 1,6-bisphosphatase genes in cancer. Biochem Biophys Res Commun. 2008;377(2):720–4. 18938139 10.1016/j.bbrc.2008.10.045

[78] Dong C, Yuan T, Wu Y, et al. Loss of FBP1 by Snail-mediated repression provides metabolic advantages in basal-like breast cancer. Cancer Cell. 2013;23(3):316–31. 23453623 10.1016/j.ccr.2013.01.022 PMC3703516

[79] Hirata H, Sugimachi K, Komatsu H, et al. Decreased expression of fructose-1,6-bisphosphatase associates with glucose metabolism and tumor progression in hepatocellular carcinoma. Cancer Res. 2016;76(11):3265–76. 27197151 10.1158/0008-5472.CAN-15-2601

[80] Samarin J, Laketa V, Malz M, et al. PI3K/AKT/mTOR-dependent stabilization of oncogenic far-upstream element binding proteins in hepatocellular carcinoma cells. Hepatology. 2016;63(3):813–26. 26901106 10.1002/hep.28357 PMC5262441

[81] Liang X, Liu X, Li W, et al. A novel variant in the FBP1 gene causes fructose-1,6-bisphosphatase deficiency through increased ubiquitination. Arch Biochem Biophys. 2023;1(742): 109619. 10.1016/j.abb.2023.109619 37142076

[82] Cheng X, Zhang B, Guo F, et al. Deubiquitination of FBP1 by USP7 blocks FBP1-DNMT1 interaction and decreases the sensitivity of pancreatic cancer cells to PARP inhibitors. Mol Oncol. 2022;16(7):1591–607. 34854226 10.1002/1878-0261.13149 PMC8978517

[83] Zhang P, Shao Y, Quan F, et al. FBP1 enhances the radiosensitivity by suppressing glycolysis via the FBXW7/mTOR axis in nasopharyngeal carcinoma cells. Life Sci. 2021;15(283): 119840. 10.1016/j.lfs.2021.119840 34298040

[84] Chen SH, Chang JY. New insights into mechanisms of cisplatin resistance: from tumor cell to microenvironment. Int J Mol Sci. 2019;20(17):4136. 31450627 10.3390/ijms20174136 PMC6747329

[85] Ai Z, Lu Y, Qiu S, et al. Overcoming cisplatin resistance of ovarian cancer cells by targeting HIF-1-regulated cancer metabolism. Cancer Lett. 2016;373(1):36–44. 26801746 10.1016/j.canlet.2016.01.009 PMC4769873

[86] Hulleman E, Kazemier KM, Holleman A, et al. Inhibition of glycolysis modulates prednisolone resistance in acute lymphoblastic leukemia cells. Blood. 2009;113(9):2014–21. 18978206 10.1182/blood-2008-05-157842 PMC4081395

[87] Farge T, Saland E, de Toni F, et al. Chemotherapy-resistant human acute myeloid leukemia cells are not enriched for leukemic stem cells but require oxidative metabolism. Cancer Discov. 2017;7(7):716–35. 28416471 10.1158/2159-8290.CD-16-0441 PMC5501738

[88] Jauset T, Massó-Vallés D, Martínez-Martín S, et al. BET inhibition is an effective approach against KRAS-driven PDAC and NSCLC. Oncotarget. 2018;9(27):18734–46. 29721157 10.18632/oncotarget.24648 PMC5922351

[89] Sahai V, Kumar K, Knab LM, et al. BET bromodomain inhibitors block growth of pancreatic cancer cells in three-dimensional collagen. Mol Cancer Ther. 2014;13(7):1907–17. 24807963 10.1158/1535-7163.MCT-13-0925 PMC4090266

[90] Andricovich J, Perkail S, Kai Y, et al. Loss of KDM6A Activates Super-Enhancers to Induce Gender-Specific Squamous-like Pancreatic Cancer and Confers Sensitivity to BET Inhibitors. Cancer Cell. 2018;33(3):512-526.e8. 29533787 10.1016/j.ccell.2018.02.003 PMC5854186

[91] Bian B, Bigonnet M, Gayet O, et al. Gene expression profiling of patient-derived pancreatic cancer xenografts predicts sensitivity to the BET bromodomain inhibitor JQ1: implications for individualized medicine efforts. EMBO Mol Med. 2017;9(4):482–97. 28275007 10.15252/emmm.201606975 PMC5376755

[92] Wirth M, Mahboobi S, Krämer OH, et al. Concepts to target MYC in pancreatic cancer. Mol Cancer Ther. 2016;15(8):1792–8. 27406986 10.1158/1535-7163.MCT-16-0050

[93] Xin B, Yamamoto M, Fujii K, et al. Critical role of Myc activation in mouse hepatocarcinogenesis induced by the activation of AKT and RAS pathways. Oncogene. 2017;36(36):5087–97. 28481866 10.1038/onc.2017.114 PMC5596209

[94] Kumar K, Raza SS, Knab LM, et al. GLI2-dependent c-MYC upregulation mediates resistance of pancreatic cancer cells to the BET bromodomain inhibitor JQ1. Sci Rep. 2015;25(5):9489. 10.1038/srep09489 PMC4452877 25807524

[95] Fryer RA, Barlett B, Galustian C, et al. Mechanisms underlying gemcitabine resistance in pancreatic cancer and sensitisation by the iMiD™ lenalidomide. Anticancer Res. 2011;31(11):3747–56. 22110196

[96] Jin X, Pan Y, Wang L, et al. Fructose-1,6-bisphosphatase Inhibits ERK Activation and Bypasses Gemcitabine Resistance in Pancreatic Cancer by Blocking IQGAP1-MAPK Interaction. Cancer Res. 2017;77(16):4328–41. 28720574 10.1158/0008-5472.CAN-16-3143 PMC5581962

[97] Li J, Dai P, Sun J, et al. FBP1 induced by β-elemene enhances the sensitivity of gefitinib in lung cancer. Thorac Cancer. 2023;14(4):371–80. 36525508 10.1111/1759-7714.14750 PMC9891864

[98] Gonda TA, Fang J, Salas M, et al. A DNA hypomethylating drug alters the tumor microenvironment and improves the effectiveness of immune checkpoint inhibitors in a mouse model of pancreatic cancer. Cancer Res. 2020;80(21):4754–67. 32816859 10.1158/0008-5472.CAN-20-0285 PMC9296074

[99] Sultana F, Manasa KL, Shaik SP, et al. Zinc dependent histone deacetylase inhibitors in cancer therapeutics: Recent update. Curr Med Chem. 2019;26(40):7212–80. 29852860 10.2174/0929867325666180530094120

[100] West AC, Johnstone RW. New and emerging HDAC inhibitors for cancer treatment. J Clin Invest. 2014;124(1):30–9. 24382387 10.1172/JCI69738 PMC3871231

[101] Zhang C, Richon V, Ni X, et al. Selective induction of apoptosis by histone deacetylase inhibitor SAHA in cutaneous T-cell lymphoma cells: relevance to mechanism of therapeutic action. J Invest Dermatol. 2005;125(5):1045–52. 16297208 10.1111/j.0022-202X.2005.23925.x

[102] Crisanti MC, Wallace AF, Kapoor V, et al. The HDAC inhibitor panobinostat (LBH589) inhibits mesothelioma and lung cancer cells in vitro and in vivo with particular efficacy for small cell lung cancer. Mol Cancer Ther. 2009;8(8):2221–31. 19671764 10.1158/1535-7163.MCT-09-0138 PMC3605895

[103] Kane RC, Farrell AT, Sridhara R, et al. United States Food and Drug Administration approval summary: bortezomib for the treatment of progressive multiple myeloma after one prior therapy. Clin Cancer Res. 2006;12(10):2955–60. 16707588 10.1158/1078-0432.CCR-06-0170

[104] Lee YB, Min JK, Kim JG, et al. Multiple functions of pyruvate kinase M2 in various cell types. J Cell Physiol. 2022;237(1):128–48. 34311499 10.1002/jcp.30536

[105] Israelsen WJ, Vander Heiden MG. Pyruvate kinase: Function, regulation and role in cancer. Semin Cell Dev Biol. 2015;43:43–51. 26277545 10.1016/j.semcdb.2015.08.004 PMC4662905

[106] Christofk HR, Vander Heiden MG, Harris MH, et al. The M2 splice isoform of pyruvate kinase is important for cancer metabolism and tumour growth. Nature. 2008;452(7184):230–3. 18337823 10.1038/nature06734

[107] Xu Q, Tu J, Dou C, et al. HSP90 promotes cell glycolysis, proliferation and inhibits apoptosis by regulating PKM2 abundance via Thr-328 phosphorylation in hepatocellular carcinoma. Mol Cancer. 2017;16(1):178. 29262861 10.1186/s12943-017-0748-y PMC5738801

[108] Dhar DK, Olde Damink SW, Brindley JH, et al. Pyruvate kinase M2 is a novel diagnostic marker and predicts tumor progression in human biliary tract cancer. Cancer. 2013;119(3):575–85. 22864959 10.1002/cncr.27611 PMC3492546

[109] Das R, Ghosh Chowdhury M, Raundal S, et al. Objective assessment of adrenocortical carcinoma driver genes and their correlation with tumor pyruvate kinase M2. Gene. 2022;15(822): 146354. 10.1016/j.gene.2022.146354 35189247

[110] Mazurek S, Boschek CB, Hugo F, et al. Pyruvate kinase type M2 and its role in tumor growth and spreading. Semin Cancer Biol. 2005;15(4):300–8. 15908230 10.1016/j.semcancer.2005.04.009

[111] Keller KE, Doctor ZM, Dwyer ZW, et al. SAICAR induces protein kinase activity of PKM2 that is necessary for sustained proliferative signaling of cancer cells. Mol Cell. 2014;53(5):700–9. 24606918 10.1016/j.molcel.2014.02.015 PMC4000728

[112] Yang W, Xia Y, Hawke D, et al. PKM2 phosphorylates histone H3 and promotes gene transcription and tumorigenesis. Cell. 2012;150(4):685–96. 22901803 10.1016/j.cell.2012.07.018 PMC3431020

[113] Gao X, Wang H, Yang JJ, et al. Pyruvate kinase M2 regulates gene transcription by acting as a protein kinase. Mol Cell. 2012;45(5):598–609. 22306293 10.1016/j.molcel.2012.01.001 PMC3299833

[114] Jiang Y, Li X, Yang W, et al. PKM2 regulates chromosome segregation and mitosis progression of tumor cells. Mol Cell. 2014;53(1):75–87. 24316223 10.1016/j.molcel.2013.11.001 PMC3955203

[115] Jiang Y, Wang Y, Wang T, et al. PKM2 phosphorylates MLC2 and regulates cytokinesis of tumour cells. Nat Commun. 2014;21(5):5566. 10.1038/ncomms6566 PMC4259466 25412762

[116] Kapoor S, Chatterjee DR, Chowdhury MG, et al. Roadmap to Pyruvate Kinase M2 Modulation - A Computational Chronicle. Curr Drug Targets. 2023;24(6):464–83. 36998144 10.2174/1389450124666230330103126

[117] Rathod B, Chak S, Patel S, et al. Tumor pyruvate kinase M2 modulators: a comprehensive account of activators and inhibitors as anticancer agents. RSC Med Chem. 2021;12(7):1121–41. 34355179 10.1039/d1md00045d PMC8292966

[118] Jadhav J, Das R, Kamble S, et al. Ferrocene-based modulators of cancer-associated tumor pyruvate kinase M2. J Organomet Chem. 2022;1: 122338.

[119] Das R, Pulugu P, Singh AA, et al. Mechanistic Investigation of Thiazole-Based Pyruvate Kinase M2 Inhibitor Causing Tumor Regression in Triple-Negative Breast Cancer. J Med Chem. 2024;67(5):3339–57. 38408027 10.1021/acs.jmedchem.3c01512

[120] Das R, Chatterjee DR, Kapoor S, et al. Novel sulfonamides unveiled as potent anti-lung cancer agents via tumor pyruvate kinase M2 activation. RSC Med Chem. 2024;15(9):3070–91. 39309364 10.1039/d4md00367e PMC11411637

[121] Chowdhury MG, Kapoor S, Muthukumar V, et al. Development of novel tetrazole-based pyruvate kinase M2 inhibitors targeting U87MG glioblastoma cells. Bioorg Chem. 2025;154: 108029. 39693922 10.1016/j.bioorg.2024.108029

[122] Hanahan D, Weinberg RA. Hallmarks of cancer: the next generation. Cell. 2011;144(5):646–74. 21376230 10.1016/j.cell.2011.02.013

[123] Giovannucci E, Harlan DM, Archer MC, et al. Diabetes and cancer: a consensus report. Diabetes Care. 2010;33(7):1674–85. 20587728 10.2337/dc10-0666 PMC2890380

[124] Wettersten HI, Aboud OA, Lara PN Jr, et al. Metabolic reprogramming in clear cell renal cell carcinoma. Nat Rev Nephrol. 2017;13(7):410–9. 28480903 10.1038/nrneph.2017.59

[125] Matsuda Y, Miura K, Yamane J, et al. SERPINI1 regulates epithelial-mesenchymal transition in an orthotopic implantation model of colorectal cancer. Cancer Sci. 2016;107(5):619–28. 26892864 10.1111/cas.12909 PMC4970828

[126] Damanakis A, Plum PS, Gebauer F, et al. Fructose-1,6-bisphosphatase 1 (FBP1) is an independent biomarker associated with a favorable prognosis in esophageal adenocarcinoma. J Cancer Res Clin Oncol. 2022;148(9):2287–93. 35477823 10.1007/s00432-022-04025-x PMC9349078

[127] Liu M, Pan Q, Xiao R, et al. A cluster of metabolism-related genes predict prognosis and progression of clear cell renal cell carcinoma. Sci Rep. 2020;10(1):12949. 32737333 10.1038/s41598-020-67760-6 PMC7395775

[128] Liu ZH, Hu JL, Liang JZ, et al. Far upstream element-binding protein 1 is a prognostic biomarker and promotes nasopharyngeal carcinoma progression. Cell Death Dis. 2015;6(10): e1920. 26469968 10.1038/cddis.2015.258 PMC4632288

[129] Gizak A, Budziak B, Domaradzka A, et al. Fructose 1,6-bisphosphatase as a promising target of anticancer treatment. Adv Biol Regul. 2024;23: 101057. 10.1016/j.jbior.2024.101057 39490352

[130] Gao X, Ren X, Wang F, et al. Immunotherapy and drug sensitivity predictive roles of a novel prognostic model in hepatocellular carcinoma. Sci Rep. 2024;14(1):9509. 38664521 10.1038/s41598-024-59877-9 PMC11045740

